# Role of DNA methylation in expression control of the *IKZF3-GSDMA* region in human epithelial cells

**DOI:** 10.1371/journal.pone.0172707

**Published:** 2017-02-27

**Authors:** Sanny Moussette, Abeer Al Tuwaijri, Hamid-Reza Kohan-Ghadr, Samar Elzein, Raquel Farias, Julie Bérubé, Bianca Ho, Catherine Laprise, Cynthia G. Goodyer, Simon Rousseau, Anna K. Naumova

**Affiliations:** 1 The Research Institute of the McGill University Health Centre, Montreal, Quebec, Canada; 2 Department of Human Genetics, McGill University Montreal, Quebec, Canada; 3 The Meakins-Christie Laboratories at the Research Institute of the McGill University Health Centre, Montreal, Quebec, Canada; 4 Département des sciences fondamentales, Université du Québec à Chicoutimi, Chicoutimi, Quebec, Canada; 5 Department of Pediatrics and Experimental Medicine, McGill University, Montreal, Quebec, Canada; 6 Department of Obstetrics and Gynecology, McGill University, Montreal, Quebec, Canada; University of South Alabama Mitchell Cancer Institute, UNITED STATES

## Abstract

Chromosomal region 17q12-q21 is associated with asthma and harbors regulatory polymorphisms that influence expression levels of all five protein-coding genes in the region: IKAROS family zinc finger 3 (Aiolos) (*IKZF3*), *zona pellucida* binding protein 2 (*ZPBP2)*, ORMDL sphingolipid biosynthesis regulator 3 (*ORMDL3*), and gasdermins A and B (*GSDMA*, *GSDMB)*. Furthermore, DNA methylation in this region has been implicated as a potential modifier of the genetic risk of asthma development. To further characterize the effect of DNA methylation, we examined the impact of treatment with DNA methyltransferase inhibitor 5-aza-2’-deoxycytidine (5-aza-dC) that causes DNA demethylation, on expression and promoter methylation of the five 17q12-q21 genes in the human airway epithelium cell line NuLi-1, embryonic kidney epithelium cell line 293T and human adenocarcinoma cell line MCF-7. 5-aza-dC treatment led to upregulation of expression of *GSDMA* in all three cell lines. *ZPBP2* was upregulated in NuLi-1, but remained repressed in 293T and MCF-7 cells, whereas *ORMDL3* was upregulated in 293T and MCF-7 cells, but not NuLi-1. Upregulation of *ZPBP2* and *GSDMA* was accompanied by a decrease in promoter methylation. Moreover, 5-aza-dC treatment modified allelic expression of *ZPBP2* and *ORMDL3* suggesting that different alleles may respond differently to treatment. We also identified a polymorphic CTCF-binding site in intron 1 of *ORMDL3* carrying a CG SNP rs4065275 and determined its methylation level. The site’s methylation was unaffected by 5-aza-dC treatment in NuLi-1 cells. We conclude that modest changes (8–13%) in promoter methylation levels of *ZPBP2* and *GSDMA* may cause substantial changes in RNA levels and that allelic expression of *ZPBP2* and *ORMDL3* is mediated by DNA methylation.

## Introduction

Genome-wide association studies (GWAS) have identified thousands of loci associated with human disease. In most cases, however, the genetic association alone cannot accurately predict whether an individual carrier of the risk allele will develop the disease. Such an uncertain heritability is explained by differences in environmental exposures or epigenetic variation between individuals [1]. Therefore, it has been suggested that inter-individual variation in epigenetic states, such as DNA methylation, may modify the risk of developing disease. Furthermore, emerging data suggest that DNA methylation may act as a mediator of the effect of genotype on gene expression or provide a mode of communication and adaptation between the genome and environment (reviewed in [2]).

Chromosomal region 17q12-q21 harbours one of the best replicated GWAS regions associated with childhood asthma [3–6]. The 17q12-q21 common polymorphisms associated with asthma delineate a genomic interval that encompasses five protein-coding genes: IKAROS family zinc finger 3 (Aiolos) (*IKZF3*), *zona pellucida* binding protein 2 (*ZPBP2*), gasdermin B (*GSDMB*), ORMDL sphingolipid biosynthesis regulator 3 (*ORMDL3*), and gasdermin A (*GSDMA*). Expression of all of these genes depends on genotype in a number of different cell types [3, 7, 8]. Two common haplotypes associated with expression levels and spanning about 160 kb were first delineated in studies of lymphoblastoid cell lines (LCLs) and termed HapA and HapB [7, 9]. Haplotype HapA is associated with higher expression levels of *ORMDL3* and *GSDMB* in cells from peripheral blood, LCLs, mammary tissue, lungs and several other tissues; *ZPBP2* in testes and *IKZF3* in the aorta [7]. Haplotype HapB (sum of all non-HapA haplotypes) is associated with higher expression of *ZPBP2* in LCLs and *GSDMA* in lungs and mammary tissue [7–10]. The HapA haplotype harbors variants associated with childhood asthma [7]. It is widely accepted that elevated expression of *ORMDL3* confers higher risk for asthma [3, 11–13]. However, IKZF3, GSDMA and GSDMB proteins are detected in human airway epithelial cells, whereas ZPBP2 appears in the glandular epithelium of the bronchus, albeit at very low levels[14]. Therefore, potential involvement of these genes in predisposition to airway disease cannot be completely ruled out [7].

Both genetic and environmental factors contribute to asthma pathogenesis; therefore, a number of studies have explored the relationship between genetic predisposition and environmental exposures. Indeed, the genetic association between 17q12-q21 alleles and asthma was stronger when exposure to tobacco smoke, farm animals and respiratory infections were taken into account [15–19]. Multiple lines of evidence suggest that DNA methylation may act as intermediary between genotype and phenotype or environment and phenotype [20–22]. In line with such a role, DNA methylation levels in the 17q12-q21 genes also show association with predisposition to asthma [1, 23]. Associations between promoter methylation and expression levels of *ZPBP2* and *GSDMB* were found in LCLs [9]. Negative association between *ORMDL3* intron 1 methylation and *ORMDL3* expression was also found in peripheral blood cells [1].

The sum of current data supports the link between DNA methylation, genotype, gene expression and asthma. However, to date, direct evidence for DNA methylation mediating or modulating the genotype effect on 17q12-q21 gene expression is scarce. Furthermore, DNA methylation differences between asthmatic and non-asthmatic subjects are rather modest (within the range of 1 to 9%) [23, 24], which often raises the question of whether such small changes in methylation levels have significant functional effects. It is also possible that differences in DNA methylation at certain regions may have no impact on gene expression.

The most parsimonious explanation for the role of DNA methylation in gene regulation is that methylation of CGs located within transcription factor (TF) binding sites interferes with their binding to DNA leading to changes in transcription. Ample experimental evidence shows that increased methylation in promoter or enhancer regions leads to gene silencing/down-regulation [25–27]. The consequences of methylation of CGs located within the insulator protein CCCTC binding factor (CTCF) binding sites (CBS) present a more complex paradigm. CTCF plays a pivotal role in the 3D organization of chromatin and gene regulation (reviewed in [28]). Methylation of CGs within the CBS prevents CTCF binding and causes dramatic changes in gene regulation due to remodeling of chromatin loops, with certain genes being upregulated and other genes repressed [29–31]. Therefore, to understand the biological processes affected by DNA methylation changes, the functions of the differentially methylated CGs have to be taken into account.

To clarify the role of DNA methylation in the regulation of the 17q12-q21 asthma-associated region, we have examined the impact of DNA-methyltransferase 1 inhibitor 5-aza-2’-deoxycytidine (5-aza-dC) on DNA methylation of regulatory elements and gene expression in 3 human epithelium cell lines: airway epithelium cell line NuLi-1, the embryonic kidney epithelium cell line 293T and the epithelial metastatic breast adenocarcinoma cell line MCF-7. We found that these different cell lines respond differently to 5-aza-dC treatment. We also observed that modest changes in DNA methylation caused a several-fold increase in the expression of *ZPBP2* and *GSDMA*, the same genes that show methylation differences between asthmatic and non-asthmatic subjects in blood cells [23], and that the allelic expression bias of *ZPBP2* and *ORMDL3* depended on methylation in NuLi-1 cells.

## Materials and methods

### Cell lines and cell culture

List of cell lines used in this study is provided in S1 Table. The human airway epithelial cell line, NuLi-1, was derived from a normal lung of a 36-year-old male patient by dual retroviral infection to prevent cells undergoing growth arrest in cell culture [32]. The CuFi-1 airway epithelial cell line was derived from the lung of a 14-year-old female patient with cystic fibrosis by the same method and is homozygous for the CFTRΔF508 mutation [32]. These cells were purchased from the ATCC. N3 (normal bronchial epithelial cells), CF2 (homozygous for the CFTRΔF508 mutation) and CF7 (CFTRG551D mutation) cells were kindly provided by Dr Scott Randell (Cystic Fibrosis/Pulmonary Research and Treatment Center, The University of North Carolina, Chapel Hill, North Carolina) [32]. All of these cells were cultured on collagen I and maintained at 37°C, 5% CO_2_, 100% humidity, in Bronchial Epithelial Growth Medium (Lonza, Walkersville, MD) supplemented with growth factors (SingleQuots (Lonza), except gentamicin), 50 U/ml penicillin G and 50 μg/ml streptomycin.

The human embryonic kidney cell line 293T was cultured in Dulbecco’s modified Eagle’s medium (DMEM, 319-006-CL; Wisent BioProducts, QC, Canada) at 37°C, 5% CO_2_, and 100% humidity. Culture medium was supplemented with heat-inactivated (10% v/v) fetal bovine serum (FBS, 080–450; Wisent Bioproducts, QC, Canada), 50 U/ml penicillin G, 50 μg/ml streptomycin. These cells were purchased from ATCC.

The human adenocarcinoma MCF-7 cells were grown in DMEM medium (Gibco, Life Technologies) supplemented with 10% FBS, 25mM HEPES, 50 U/ml penicillin and 1.6 mg/ml gentamycin. Cells were incubated at 37°C, 5% CO_2_, and 100% humidity. These cells were purchased from ATCC.

5-aza-2’-deoxycytidine (5-aza-dC) (Sigma-Aldrich, USA) was dissolved in DMSO at 50 mg/ml. NuLi-1 cells (passages 17–21) were plated in 60 × 15 mm Petri dishes (1.5 x 10^5^ cells per plate) and treated with 0.5 μM 5-aza-dC in 0.5% DMSO or 0.5% DMSO alone for 24h. After 24h, the medium was changed and cells were grown for another 7 days. Cells were harvested for RNA and DNA extraction. Effect of 5-aza-dC on expression was evaluated in 4, and the effect on DNA methylation—in 3 independent replicate experiments. A similar treatment was done for MCF-7 cells in 3 replicate experiments. The most efficient treatment for 293T cells was at 50 μM 5-aza-dC concentration. After initial 5-aza-dC treatment, 293T cells were cultured for 3 days. The 293T experiment was done in triplicate. Imprinted gene *H19* whose expression increases with promoter demethylation was used as control for treatment efficiency.

### Genotyping

Epithelial cell line 17q12-q21 genotypes were established using PCR assays targeting SNPs in the *ZPBP2* and *ORMDL3* genic regions followed by Sanger sequencing. Sequencing was done by the McGill University and Genome Quebec Innovation Centre sequencing service. PCR primers are listed in S2 Table.

### Expression analysis and FAIRE assays

RNA was extracted using Trizol reagent (Life Technologies) and purified using RNeasy MinElute Cleanup Kit (Qiagen). Quantitative real-time RT-PCR (qPCR) was performed using Power SYBR^R^ Green PCR Master mix (Applied Biosystems, San Diego, CA, USA) in the Eco^™^ Real-Time PCR System (Illumina, San Diego, CA, USA). Data were normalized by 18S RNA levels and analyzed using the 2(-delta-delta C(T)) method [33]. Statistical significance of expression differences between 5-aza-dC treated cells and controls was evaluated using the Student’s t-test.

Allelic expression of genes from the human chromosomal region 17q12-q21 was analyzed using RT-PCR followed by Sanger sequencing. For each of the analyzed genes, primers targeting exonic SNPs were selected. Primers and targeted SNPs are listed in S2 Table. Sequencing was done by the McGill University and Genome Quebec Innovation Centre sequencing service. Allelic ratios were determined using the PeakPicker software [34].

DNA from previously described FAIRE assays [7, 9] was used to determine the effect of SNP rs4065275 on nucleosome occupancy.

### Sodium bisulfite methylation assays

Sodium bisulfite sequencing DNA methylation assays for *ZPBP2*, *ORMDL3*, and *GSDMA* promoters were conducted as previously described [9, 35]. On average, 24 clones per sample were sequenced. Clone sequences were analyzed and the average percent of methylation was calculated for the region (number of methylated CG vs. number of assayed CGs) and for each individual CG position.

Pyrosequencing assays for *ZPBP2* and *GSDMA* were conducted as previously described [23, 35]. The pyrosequencing assay for the CTCF sites C9a and C9b located within intron 1 of *ORMDL3* was designed with PyroMark Assay Design 2.0 software. Bisulfite conversion was done using the EpiTect bisulfite kit (Qiagen). The pyrosequencing assay was performed with PyroMark Q24 Advanced CpG Reagents (Qiagen) in the PyroMark Advanced Q24 system. Percentage of DNA methylation per CG site was analyzed with PyroMark Q24 Advanced 3.0.0 software. Statistical significance of the change in methylation was evaluated using the Student’s t-test.

## Results

### DNA methyltransferase 1 inhibitor 5-aza-2’-deoxycytidine treatment alters gene expression in the 17q12-q21 region

**Fig 1:**
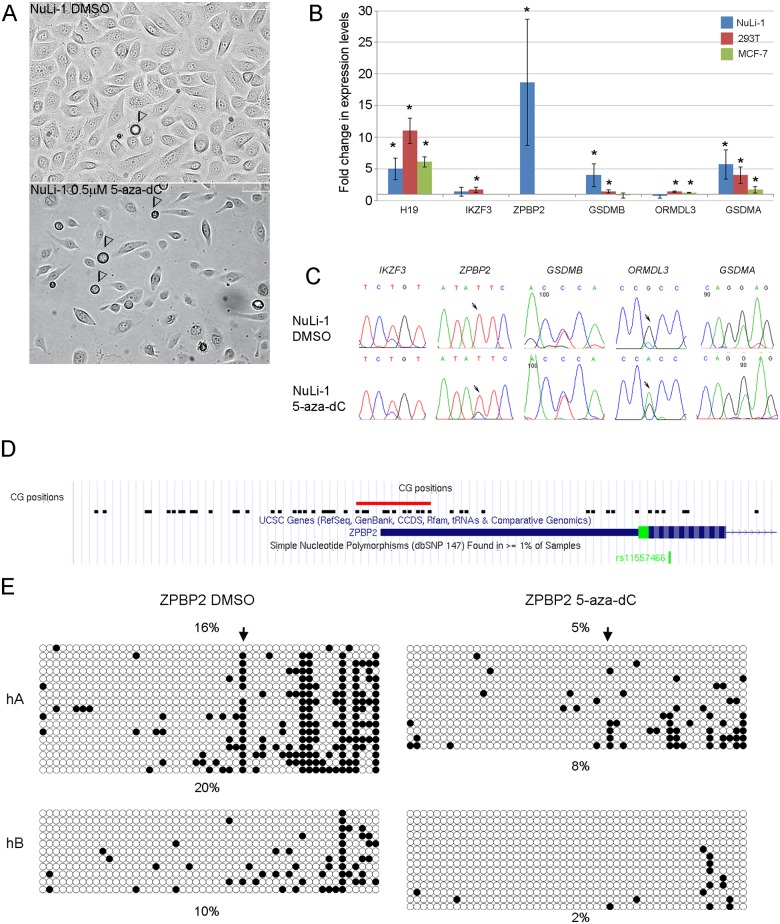
5-aza-dC treatment enhances gene expression. (A) 5-aza-dC treated NuLi-1 cells show reduced proliferation and apoptosis four days after treatment. Arrowheads point to dying/dead cells. (B) Changes in expression levels of 17q12-q21 genes after 5-aza-dC treatment. The y-axis shows fold change in 5-aza-dC treated cells compared to controls. Error bars show standard deviation. Asterisks indicate statistically significant change in expression in 5-aza-dC treated cells compared to controls (* p < 0.05). (C) Allelic expression in 17q12-q21 genes after 5-aza-dC treatment. Arrows show positions of transcribed SNPs in those genes where allelic expression changed post 5-aza-dC treatment. In *ZPBP2*, 5-aza-dC treatment causes reactivation of the HapA allele. In *ORMDL3* it causes a switch in allelic preference. (D) Positions of 51 CGs in the *ZPBP2* promoter region that were assayed using the sodium bisulfite sequencing assay are shown in the context of the UCSC browser. The red box indicates the position of the 11 CGs assayed using the pyrosequencing methylation assay. (E) DNA methylation profiles of the *ZPBP2* promoter region in control (DMSO) and 5-aza-dC treated cells. Filled circles represent methylated CGs, open circles represent unmethylated CGs. Each row represents a clone. Data are divided by haplotype; allelic percent methylation is shown below the diagram. Type of treatment and average methylation levels are shown on top. Arrow points to CG31 (CG6 in pyrosequencing assays) that has one of the most pronounced allelic differences in methylation.

To determine the impact of 5-aza-dC, we examined expression levels and allelic expression of five 17q12-q21 genes in NuLi-1, 293T and MCF-7 cells (S1 Table). In all 3 cell lines, treatment with 5-aza-dC led to inhibition of cell proliferation and upregulation of *H19* expression (Fig 1A and 1B). Expression levels of *ORMDL3* were not affected in NuLi-1, but increased 1.2 and 1.4-fold in MCF-7 and 293T cells, respectively (Fig 1B). *GSDMA* was upregulated in all three cell lines. *GSDMB* was upregulated in NuLi-1 and 293T cells, with no significant change in MCF-7 cells (Fig 1B). *ZPBP2* was expressed only in the NuLi-1 cell line before treatment and was upregulated almost 20-fold, but remained silent in 293T and MCF-7 cells. *IKZF3* was expressed in NuLi-1 and 293T cells; it was upregulated in 293T but not NuLi-1 cells post 5-aza-dC treatment.

Allelic expression analysis was possible only in NuLi-1 cells that are heterozygous for the *IKZF3-GSDMA* genomic interval; 293T and MCF-7 cells are homozygous and therefore not informative. In control NuLi-1 cells, allelic bias was detected in the expression of *IKZF3* and *ZPBP2* in favor of the HapB alleles, *ORMDL3* and *GSDMA* in favor of the HapA alleles; *GSDMB* was expressed equally from both alleles. 5-aza-dC treatment caused a switch in the allelic bias of *ORMDL3* gene expression from the HapA to the HapB allele. It also reactivated the silent *ZPBP2* HapA allele making the *ZPBP2* allelic bias in favor of the HapB allele less extreme (Fig 1C).

### 5-aza-dC treatment changes DNA methylation at *ZPBP2* and *GSDMA* promoters

**Fig 2:**
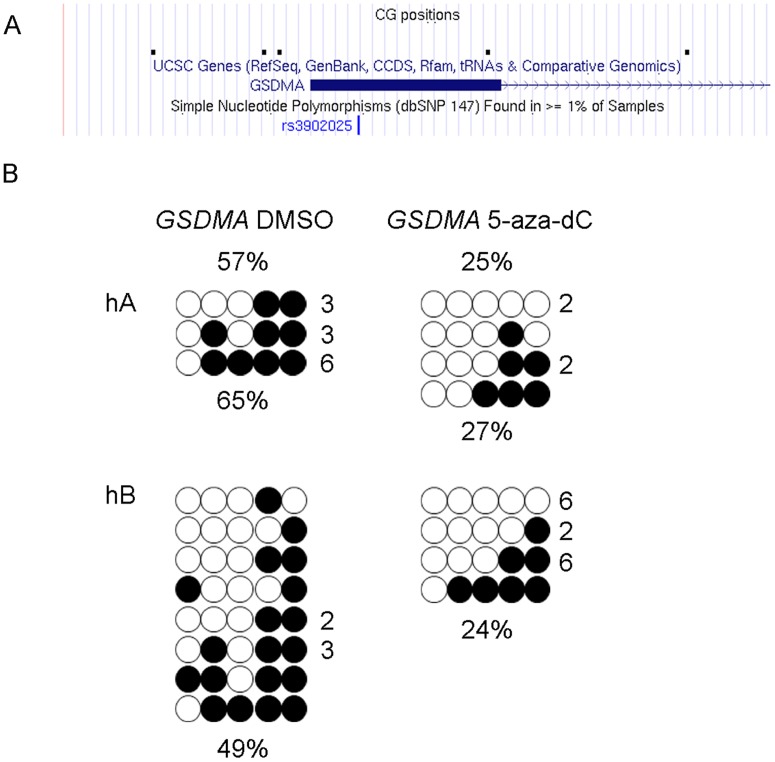
DNA methylation patterns of the *GSDMA* promoter in NuLi-1 cells. (A) Positions of the interrogated CGs with respect to the *GSDMA* promoter region are shown in the context of the UCSC browser. (B) *GSDMA* promoter methylation changes after 5-aza-dC treatment. Filled circles represent methylated CGs, open circles represent unmethylated CGs. Each row represents a clone, the number on the right indicates the number of clones with a particular methylation pattern. Data are divided by allele; allelic percent methylation is shown below the diagram. Type of treatment and average DNA methylation are shown on top; hA -haplotype A; hB–haplotype B.

The most pronounced effect of 5-aza-dC on expression levels was seen for *ZPBP2* in NuLi-1 and for *GSDMA* in all three cells lines. To determine if up-regulation of *ZPBP2* and *GSDMA* expression was due to promoter demethylation, we conducted DNA methylation assays in NuLi-1 and 293T cells. After treatment with 5-aza-dC, the average methylation level of the *ZPBP2* promoter was reduced to 5% from 16% in NuLi-1 cells as determined using the sodium bisulfite sequencing methylation assay (Fig 1E). Loss of methylation was confirmed using the pyrosequencing methylation assay (S3 Table). In 293T cells, the *ZPBP2* promoter was hypermethylated and treatment had very little effect on its methylation level (64.3% vs 62.3%, control *vs* treatment). Average methylation of the *GSDMA* promoter was reduced to 25% from 57% in NuLi-1 cells and to 61% from 74% in 293T cells. The methylation of *GSDMA*-CG1, which shows lower methylation in asthmatic females [23], was reduced to 0% from 9% in NuLi-1 and to 23% from 31% in 293T cells. DNA from treated MCF-7 cells was not available for analysis. Thus, for *ZPBP2* and *GSDMA* promoters, reducing methylation by as little as 8% or 13% was accompanied by a marked increase in expression (Figs 1 and 2, and S3 Table).

To determine if changes in *ZPBP2* allelic bias in NuLi-1 cells was also the result of demethylation, we analyzed allelic methylation levels (Fig 1E). The HapB allele of the *ZPBP2* promoter was less methylated than the HapA allele in both controls and 5-aza-dC treated cells (10% vs 20% methylation in controls, and 2% vs 8% in treated cells, respectively) (Fig 1E). It is worth noting that CG31, one of the 5 CGs (31, 40, 41, 48, 51) which showed the largest differences between alleles as well as the biggest loss of methylation after 5-aza-dC treatment, resides near the transcriptional start site of *ZPBP2*. The decrease in CG31 methylation on the HapA allele to 36% from 88% may explain upregulation of *ZPBP2* expression from this allele after 5-aza-dC treatment.

To determine whether changes in the allelic expression bias of *ORMDL3* resulted from promoter demethylation, its methylation was also assayed. The *ORMDL3* promoter was hypomethylated in NuLi-1 5-aza-dC treated and control cells (S1 Fig). Therefore, we hypothesized that changes in allelic expression of *ORMDL3* resulted from changes in DNA methylation at another regulatory region, such as CTCF-binding site.

### Polymorphic and DNA-methylation sensitive CTCF binding sites in the *IKZF3-GSDMA* region

**Fig 3:**
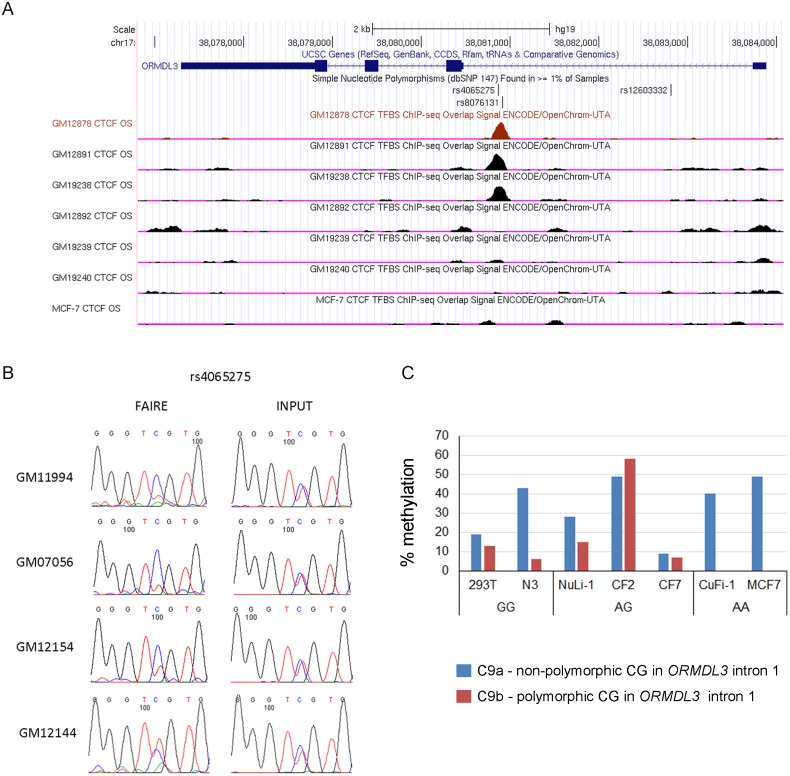
Polymorphic CTCF binding site rs4065275 (C9b) shows genotype dependent CTCF-binding, FAIRE enrichment and variable methylation levels. (A) Effect of genotype on CTCF-enrichment at the rs4065275 SNP region. Cell lines that carry the rs4065275-G allele show CTCF enrichment, whereas cell lines that carry the rs4065275-A allele do not. (B) Allelic bias in FAIRE enrichment in LCLs. In 3 out of 4 cell lines the rs4065275-G (C in the diagram) is enriched in the nucleosome-free fraction. (C) Methylation levels of the two putative CBS within the C9 CTCF-enriched region, rs4065275 CG (C9b) and the adjacent non-polymorphic CG (C9a), in human epithelial cells were determined using a pyrosequencing methylation assay. Name of cell line and rs4065275 genotype are shown below the x-axis.

The *IKZF3-GSDMA* region harbors 12 CTCF-enriched regions identified by ChIP-seq [36] (S2 Fig). We used the CTCF motif prediction tool (http://insulatordb.uthsc.edu [37]) to identify the CTCF binding motifs and determine if there were common SNPs that occurred in the CBS and that could change CTCF binding in these 12 CTCF enriched regions and two additional regions, C5 and C6, located within the *ZPBP2* promoter region (S4 Table). Two of the predicted CBS contained SNPs within the CTCF-binding motif: rs4065275 in intron 1 of *ORMDL3* (CBS C9b) and the previously reported rs12936231 in intron 5 of *ZPBP2* (CBS C7a) [7] (Fig 3 and S4 Table). To validate our predictions, we examined the CTCF ChIP-seq Uniform Peaks from ENCODE/Analysis ENCODE (March 2012 Freeze) [36] for genotype-dependent CTCF enrichment. Genotype data were available for HapMap LCLs and obtained from the HapMap database [38]. Indeed, three cell lines GM12891, GM12878 and GM19238 that were homozygous or heterozygous for the rs4065275-G allele had higher CTCF-enrichment compared to rs4065275-A homozygous cell lines GM12892, GM19239, GM19240 and MCF-7 in the C9 region (Fig 3A).

CTCF binding is usually associated with nucleosome repositioning and lower nucleosome occupancy that may be detected by formaldehyde-assisted identification of regulatory elements (FAIRE) assays [39]. Therefore, allelic bias in FAIRE enrichment at the rs4065275 region in *ORMDL3* was tested using heterozygous LCLs. The CBS C7b region in *ZPBP2* was used as control (data not shown). The CBS C9b region showed allelic differences in FAIRE enrichment in three of the 4 assayed LCLs with higher enrichment of the rs4065275-G (C on the reverse strand) allele corresponding to the HapA haplotype (Fig 3B). Hence, our data confirm that rs4065275 modifies nucleosome occupancy in an allele-specific fashion.

Within the C9 CTCF-enriched region of *ORMDL3*, the predicted non-polymorphic CBS C9a and the rs4065275-G allele of the polymorphic CBS C9b contain CG sites. To determine if methylation of these two sites varied between epithelial cell lines, pyrosequencing methylation assays were conducted in 293T, NuLi-1 and MCF-7 cells as well as CF2, CF7, N3 and CuFi-1 cell lines derived from human airway epithelium. Methylation levels ranged between 9% and 49% in C9a and between 6% and 58% in C9b (Fig 3C). This variability is higher than observed in human peripheral blood cells [1].

To determine if the allelic effect of 5-aza-dC treatment on *ORMDL3* allelic expression was due to change in C9b-CG methylation, we analyzed methylation of CBS C9a and C9b in the heterozygous NuLi-1 cells. There was no significant change in the C9b CG methylation level (S3 Table). The C9a CG methylation was reduced to 16% from 21% upon 5-aza-dC treatment, but the change was also not statistically significant (S3 Table). Since the polymorphic CBS C9b was hypomethylated and its methylation level did not show significant change, it is unlikely that this CG is responsible for the allelic bias in *ORMDL3* transcription.

## Discussion

Our previous work demonstrated that lower methylation levels of certain CGs in the *ZPBP2* and *GSDMA* promoters in peripheral blood cells are associated with asthma in females [23]. Here, we demonstrate that a modest decrease in average promoter methylation levels of these two genes causes dramatic upregulation of their transcription (4 to 20-fold increase). Therefore, differences in promoter methylation levels that were found between asthmatic and non-asthmatic females [23] are likely to be functionally relevant.

Interestingly, in our experiments, none of the genes that were silent in control cells became transcriptionally active after 5-aza-dC treatment. This may be due to a limited efficiency of 5-aza dC in heterochromatic regions. Moreover, in 293T and MCF-7 cells, we observed very modest, if any, up-regulation of G*SDMB*, whereas in NuLi-1 cells *GSDMB* was upregulated 4-fold. Perhaps the lower methylation levels and open chromatin that is permissive for transcription in the *ZPBP2* region in NuLi-1 cells facilitates the effect of 5-aza-dC on the neighboring *GSDMB*.

To determine if DNA methylation was a mediator of the effect of genotype on gene expression levels or genotype effects were independent from methylation levels, we examined allelic expression of 17q12-q21 genes before and after 5-aza-dC treatment. Two genes showed changes in allelic expression ratios, *ZPBP2* and *ORMDL3*. The other two genes with allelic bias in expression, *IKZF3* and *GSDMA*, maintained their allelic bias independent from an increase in overall expression levels. Allelic expression bias is largely attributed to SNPs altering transcription factor binding sites and is associated with a specific chromatin signature [7, 40–42]. DNA methylation has a pivotal role in controlling monoallelic expression of imprinted genes. Our data suggest that, for certain non-imprinted genes, DNA methylation is responsible for allelic expression, which is consistent with previous reports of complex relationships between DNA methylation and expression [43].

Changes in DNA methylation of different regulatory elements, such as promoters, enhancers and insulators, impact gene expression. The *ORMDL3* promoter is hypomethylated in all three tested cell lines and, hence, cannot be demethylated. However, *ORMDL3* was upregulated in 293T and MCF-7 cells, whereas, in NuLi-1 cells, expression levels remained unchanged but its allelic bias in expression was reversed towards the HapB allele. This suggested that another regulatory region was involved which led us to test the hypothesis that allelic expression of *ORMDL3* depended on methylation of a polymorphic CTCF-binding site.

Here we report that the common CG SNP rs4065275 located within a CBS modifies nucleosome positioning in such a way that the rs4065275-G allele is enriched in nucleosome-free DNA. Unlike the rs12936231 polymorphic CBS, the rs4065275 polymorphic site does not disrupt the CTCF-binding motif, but changes CTCF-enrichment *in vivo*. It is worth noting that rs4065275 has been implicated in predisposition to asthma, regulation of *ORMDL3* expression levels and TF binding in peripheral blood mononuclear cells and *in vitro* assays [44]. One would therefore expect that the unmethylated rs4065275 CG site would enhance CTCF-binding, whereas the hypermethylated would reduce it. Our data suggest that methylation of the rs4065275-associated CG site is highly variable in epithelial cell lines, but not sensitive to 5-aza-dC treatment in the NuLi-1 cells.

In summary, we find that the response to 5-aza-dC, a chemical that is the basis for anti-cancer drug decitabine, varies among different cell lines. These data suggest that the same environmental trigger may elicit different phenotypic responses from different individuals at the levels of DNA methylation and gene expression. It is also worth noting that the NuLi-1, 293T and MCF-7 cell lines assayed in our study are derived from different tissue sources and have different modal chromosome numbers. Furthermore, NuLi-1 cells are derived from a male donor, whereas 293T and MCF-7 are from female donors. Therefore, karyotype, sex, and cell type may be critical factors when it comes to response to demethylation. Further studies are necessary to determine to what extent local genotype, sex and cell type account for such a variance.

## Supporting information

S1 Table Cell line characteristics. (DOCX)

S2 Table List of primers. (DOCX)

S3 Table Effect of 5-aza-dC on methylation of the *ZPBP2* promoter and *ORMDL3* CTCF site C9 in NuLi-1 cells. Data are from pyrosequencing methylation assays of three independent 5-aza-dC treatment experiments.
(DOCX)

S4 Table Predicted CTCF-binding sites (CBS) within the CTCF-enriched regions of chromosomal region 17q12-q21 using the CTCF motif prediction tool (http://insulatordb.uthsc.edu (37)). (PDF)

S1 Fig DNA methylation of the *ORMDL3* promoter in NuLi-1 cells. Filled circles represent methylated CGs, open circles represent unmethylated CGs, gray circles represent sequencing errors. Each row represents a clone, the number on the right indicates the number of clones with a particular methylation pattern. Percent methylation is shown below the diagram. Type of treatment is shown at the right. The position of the *ORMDL3* exon 1 is shown on top.
(TIF)

S2 Fig Map of putative CTCF-binding sites in the 17q12-q21 region. Top panel. Data from the Transcription Factor ChIP-seq Uniform Peaks from ENCODE/Analysis are shown in the context of the UCSC browser (https://genome.ucsc.edu). The putative CTCF-binding regions IDs are shown at the bottom. Polymorphic CTCF binding sites shown in red. Bottom panel: the *ZPBP2* promoter region and location of putative CTCF binding sites. ID numbers correspond to those in S4 Table.
(TIF)
