# Supplementary material for: Role of DNA methylation in expression control of the IKZF3-GSDMA region in human epithelial cells
Source: PLoS One. 2017 Feb 27;12(2):e0172707. doi: 10.1371/journal.pone.0172707 (PMC5328393; doi:10.1371/journal.pone.0172707)
Supplement: S1 Table — (DOCX) [file pone.0172707.s001.docx]

**S1 Table. Cell line characteristics**

| *cell line* | *cell type* | *source* | *diagnosis* | *sex* | *age* | *immortalization* | *karyotype* | *# of chr.17* | *genotype at 17q12-q21* |
| --- | --- | --- | --- | --- | --- | --- | --- | --- | --- |
| 293T | epithelial | embryonic kidney | normal | F | fetus | SV40 | 64, XXXisoXq, multiple anomalies | 4 | HapA |
| MCF-7 | epithelial | breast cancer | adenocarcinoma | F | 69 |  | 82, XX, range 66-87, nullisomy 20 | unknown | HapB |
| NuLi-1 | epithelial | lung | normal | M | 36 | HPV,  hTERT-lxsn | 46, XY, 24% of cells are polyploid, trisomy 5 and 20 | 2 | HapAB |
