# Supplementary material for: Role of DNA methylation in expression control of the IKZF3-GSDMA region in human epithelial cells
Source: PLoS One. 2017 Feb 27;12(2):e0172707. doi: 10.1371/journal.pone.0172707 (PMC5328393; doi:10.1371/journal.pone.0172707)
Supplement: S2 Table — (DOCX) [file pone.0172707.s002.docx]

**S2 Table. List of primers**

| ***Gene*** | ***Assay*** | ***Targeted SNP*** | ***Forward primer 5'-3'*** | ***Reverse primer 5'-3'*** |
| --- | --- | --- | --- | --- |
| *18S* | qPCR | - | TGTGGTGTTGAGGAAAGCAG | GGACCTGGCTGTATTTTCCA |
| *GSDMA* | Allelic expression | rs7212944 | AGTGGAGGGAGATGTGGATG | CCTTCAGGAATGGGTGGTCT |
| *GSDMA* | qPCR | - | AAGCTGCTGGTGAAATCCAT | GGGGGAAAACACCCTCTTTA |
| *GSDMB* | Allelic expression | rs2305480 | CAAGGACCTGACAGAGGAGA | CTTCTACCAAGACCCCAGCA |
| *GSDMB* | qPCR | - | TGAAGAGCAGCAGTTTGTGG | CAGCTCATCCCAGTTCTGC |
| *H19* | qPCR | - | TGAGCTCTCAGGAGGGAGGATGGT | TTGTCACGTCCACCGGACCTG |
| *IKZF3* | qPCR | - | ACTGCACACAGGGGAAAAAC | TTGTGCTCCTCAAGGGAACT |
| *IKZF3* | Allelic expression | rs907092 | ATTCATTGGTGAGAAGCGCCA | GTGAATCGTGAACATCACATAG |
| *ORMDL3* | qPCR | - | GCAGCCAAAGCACTTTAACC | AAAAGCCTGGGACTTGGATT |
| *ORMDL3iso2* | Allelic expression/ Genotyping | rs12603332 | GAGGGGGAAAGAGCTACCAG | ACTGGCCCCTACCCTAGATG |
| *ORMDL3 CTCF sites* | Pyrosequencing | rs4065275 | GGTTGGGATAAATAATAGGTTGT^*^ | AACCTCAAAATAATTCATCCACTACT |
|  |  |  |  | CTTCAATAAAACACAATCCA^§^ |
| *ZPBP2* | Allelic expression | rs11557467 | TTTTTGGAGCCTTTGTCTGG | TGTATGAGGCCATGTTCTGG |
| *ZPBP2* | qPCR | - | GTAGTACGTCTGGATAGCTGTCG | CGCAGGTCTGACAAGTTACAT |
| *ZPBP2* | Genotyping | rs12936231 | CTTACATTAGCCCCCAGATG | TGCAGGCACATGTTTAGTCC |

* Biotinylated primer

§ Sequencing primer
