# Supplementary material for: Role of DNA methylation in expression control of the IKZF3-GSDMA region in human epithelial cells
Source: PLoS One. 2017 Feb 27;12(2):e0172707. doi: 10.1371/journal.pone.0172707 (PMC5328393; doi:10.1371/journal.pone.0172707)
Supplement: S3 Table — Data are from pyrosequencing methylation assays of three independent 5-aza-dC treatment experiments. (DOCX) [file pone.0172707.s003.docx]

**S3 Table. Effect of 5-aza-dC on methylation of the *ZPBP2* promoter and *ORMDL3* CTCF site C9 in NuLi-1 cells.**

| **Region** | **CG** | **Average methylation level in DMSO-treated cells** | **Average methylation level in**  **5-aza-dC treated cells** | ***p-value* (t-test)** | **Corresponding CG# in the SBS asssay** |
| --- | --- | --- | --- | --- | --- |
|  |  |  |  |  |  |
| ***ZPBP2*** | 1 | 9 | 10 | 0.4514 | 36 |
|  | 2 | 19 | 25 | 0.1201 | 35 |
|  | 3 | 11 | 12 | 0.3976 | 34 |
|  | 4 | 10 | 8 | 0.1189 | 33 |
|  | 5 | 8 | 5 | 0.0343 | 32 |
|  | 6 | 36 | 26 | 0.0035 | 31 |
|  | 7 | 10 | 6 | 0.0197 | 30 |
|  | 8 | 11 | 9 | 0.0237 | 29 |
|  | 9 | 11 | 9 | 0.0792 | 28 |
|  | 10 | 7 | 5 | 0.0659 | 27 |
|  | 11 | 11 | 8 | 0.0669 | 26 |
| ***ORMDL3*** | C9a | 21 | 16 | 0.0910 | n/a |
|  | C9b | 10 | 8 | 0.2829 | n/a |

Data are from pyrosequencing methylation assays of three independent 5-aza-dC treatment experiments. P-values below 0.05 are underlined.

n/a - not applicable
