# Supplementary material for: Role of DNA methylation in expression control of the IKZF3-GSDMA region in human epithelial cells
Source: PLoS One. 2017 Feb 27;12(2):e0172707. doi: 10.1371/journal.pone.0172707 (PMC5328393; doi:10.1371/journal.pone.0172707)
Supplement: S4 Table — (PDF) [file pone.0172707.s004.pdf]

S4 Table. Predicted CTCF-binding sites (CBS) within the CTCF-enriched regions of chromosomal region 17q12-q21 using the CTCF motif prediction tool (http://insulatordb.uthsc.edu (37)) .

SITE	CBS predictions	GENE	SNP variant	coordinates (hg19)	predicted CBS sequence	Position with respect to gene	Common SNPs	CG	score
C1	C1	IKZF3		chr17:37910277-37910873	CCCCACGAGGGGACG	3’	no	2	8.6
	C1	IKZF3		chr17:37910277-37910873	CAGTGACACCCAGCGGCCG	3’	no	2	6
	C1	IKZF3		chr17:37910277-37910873	GGCACTTCC	3’	no	0	10.1
C2	C2a	IKZF3		chr17:37912631-37912762	CCCT**CG**GGTGGC	3’	no	1	13.9
	C2b	IKZF3		chr17:37912631-37912762	GTTGCCTGCAGGTGGAGGGT	3’	no	0	14.4
C3	C3a	IKZF3		chr17:37991376-37991576	GAGTAGCCCCTGGTGG	gene	no	0	8.6
	C3b	IKZF3		chr17:37991376-37991576	CTTCCTGTAGGTGG**CG**ATCT	gene	no	1	14.7
	C3b	IKZF3		chr17:37991376-37991576	CTTCCTGTAGGTGG**CG**ATCT	gene	no	1	14.2
C4	C4	IKZF3		chr17:38020524-38020701	CGGAGTGCC	promoter	no	1	3.2
C5	C5	ZPBP2		chr17:38024281-38024678	GCAG**CG**CC	promoter	no	1	6.5
C6	C6	ZPBP2		chr17:38024281-38024678	CTCACAGGAGGTGGGGCTCC	Intron 1	no	0	8.7
C7	C7a	ZPBP2	rs12936231 C	chr17:38029036-38029181	GCCCCCAGATGCAG	Intron 5	rs12926321	0	2.6
	C7b	ZPBP2		chr17:38029036-38029181	TGTAGTTAC	Intron 5	no	0	7.6
	C7a	ZPBP2	rs12936231 G	chr17:38029036-38029181	TAGCCCCCAGATGGAGTGAA	Intron 5	rs12926321	0	9.8
	C7a	ZPBP2	rs12936231 G	chr17:38029036-38029181	GCCCCCAGATGGAG	Intron 5	rs12926321	0	16.4
C8	C8	GSDMB		chr17:38073333-38073599	CCCCCACCAGATGGAA	PROMOTER	no	0	15.8
	C8	GSDMB		chr17:38073333-38073599	TCCCCCACCAGATGGAAGCA	PROMOTER	no	0	13.7
C9	C9a	ORMDL3		chr17:38080703-38081095	TGAGGACCCTCTGC**CG**ACAA	gene	no	1	7.3
	C9b	ORMDL3	rs4065275 A	chr17:38080703-38081095	CAACCCCTGCTGGA	gene	rs4065275	0	10.6
	C9c	ORMDL3		chr17:38080703-38081095	GTCACTGCA	gene	no	0	8.9
	C9b	ORMDL3	rs4065275 G	chr17:38080703-38081095	**CG**ACCCCTGCTGGA	gene	rs4065275	1	14.3
	C9b	ORMDL3	rs4065275 T	chr17:38080703-38081095	CTACCCCTGCTGGA	gene	rs4065275	0	11.6
	C9b	ORMDL3	rs4065275 T	chr17:38080703-38081095	AGCACTACC	gene	rs4065275	0	10
C10	C10a	ORMDL3		chr17:38082907-38083137	AGCACCACCAGGGCAG	intron 1	no	0	7.2
	C10b	ORMDL3		chr17:38082907-38083137	TGTCTTCTGGAAGA	intron 1	no	0	3.1
C11	C11a	LRRC3C		chr17:38095884-38096115	CAGCCCCTAGTGGG	upstream	no	0	5.8
	C11a	LRRC3C		chr17:38095884-38096115	TCTGCAGCCCCTAGTGGGCA	upstream	no	0	9
	C11b	LRRC3C		chr17:38095884-38096115	TGCAGCTCC	upstream	no	0	9.5
C12	C12a	GSDMA		chr17:38115203-38115531	ACCACCAGAGGGAG	upstream	no	0	16
	C12a	GSDMA		chr17:38115203-38115531	TTGACCACCAGAGGGAGGTAG	upstream	no	0	22
	C12b	GSDMA		chr17:38115203-38115531	AGCAGCAC	upstream	no	0	5
C13	C13	PSMD3		chr17:38136189-38136443	CTGCCAGTAGGGGCTCCCAC	promoter	no	0	2.2
C14	C14a	PSMD3		chr17:38136189-38136443	GGCTGTACC	promoter	no	0	7.7
	C14b	PSMD3		chr17:38136189-38136443	GGACCTCT**CG**TGGA	promoter	no	1	4.8
