# Supplementary figures and images for: Role of DNA methylation in expression control of the IKZF3-GSDMA region in human epithelial cells
Source: PLoS One. 2017 Feb 27;12(2):e0172707. doi: 10.1371/journal.pone.0172707 (PMC5328393; doi:10.1371/journal.pone.0172707)

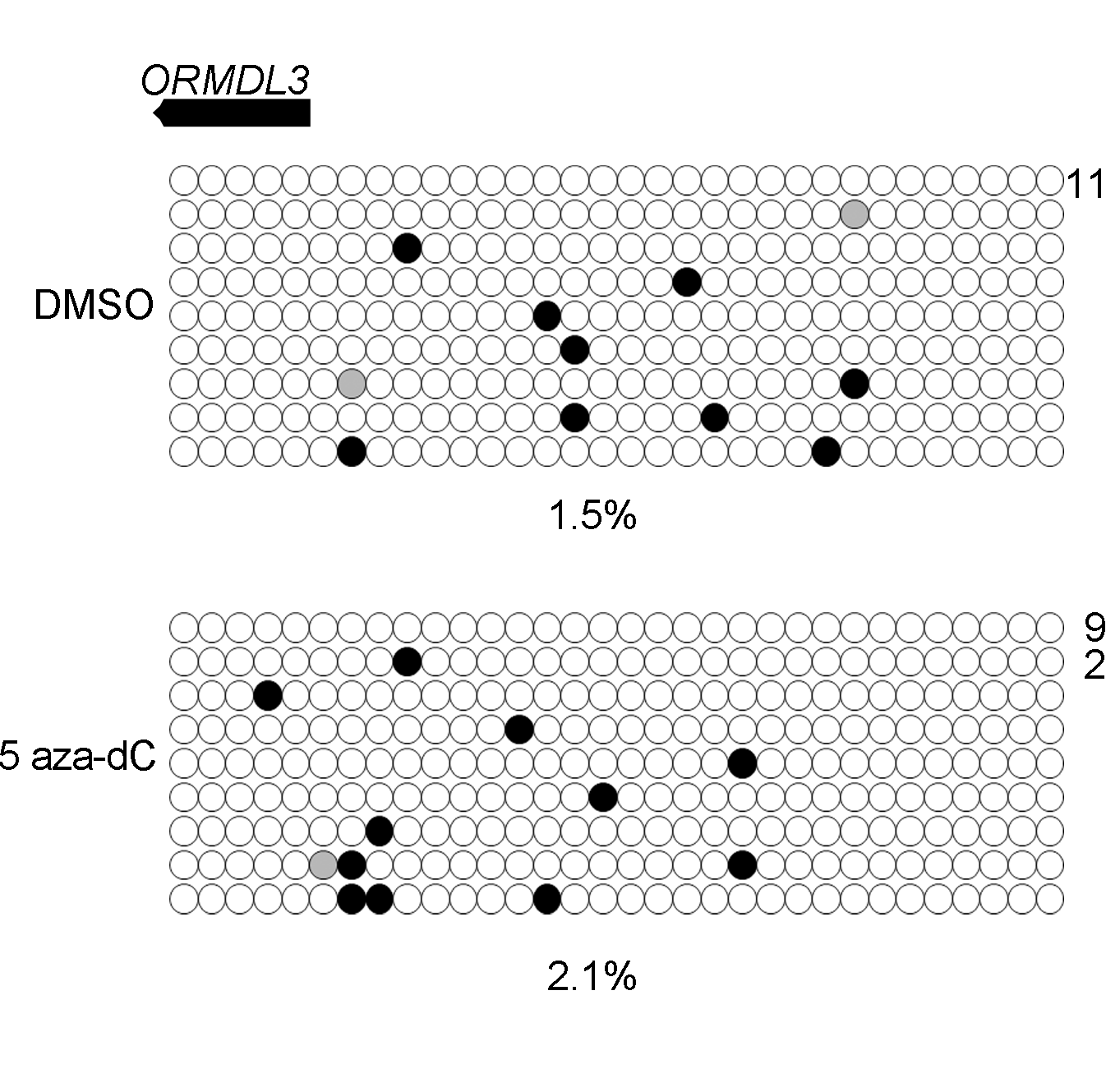

Supplement: S1 Fig — Filled circles represent methylated CGs, open circles represent unmethylated CGs, gray circles represent sequencing errors. Each row represents a clone, the number on the right indicates the number of clones with a particular methylation pattern. Percent methylation is shown below the diagram. Type of treatment is shown at the right. The position of the ORMDL3 exon 1 is shown on top. (TIF) [file pone.0172707.s005.tif]

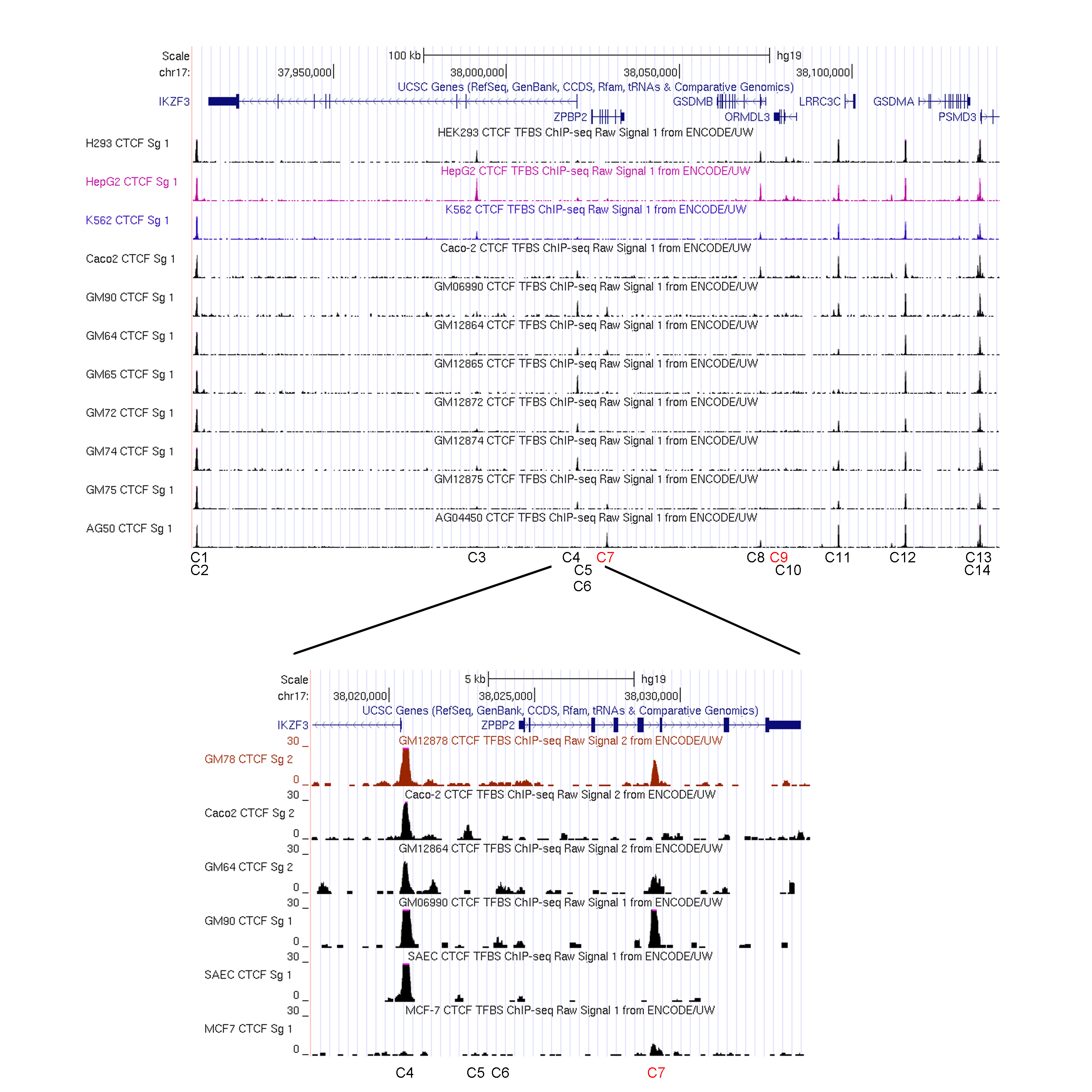

Supplement: S2 Fig — Top panel. Data from the Transcription Factor ChIP-seq Uniform Peaks from ENCODE/Analysis are shown in the context of the UCSC browser (https://genome.ucsc.edu). The putative CTCF-binding regions IDs are shown at the bottom. Polymorphic CTCF binding sites shown in red. Bottom panel: the ZPBP2 promoter region and location of putative CTCF binding sites. ID numbers correspond to those in S4 Table. (TIF) [file pone.0172707.s006.tif]
